# Image-Derived Phenotyping Informed by Independent Component Analysis—An Atlas-Based Approach

**DOI:** 10.3389/fnins.2020.00118

**Published:** 2020-02-21

**Authors:** Mahdi Moradi, Hamed Ekhtiari, Teresa A. Victor, Martin Paulus, Rayus Kuplicki

**Affiliations:** ^1^Laureate Institute for Brain Research, Tulsa, OK, United States; ^2^Department of Computer Science, University of Tulsa, Tulsa, OK, United States; ^3^Department of Community Medicine, Oxley College of Health Sciences, University of Tulsa, Tulsa, OK, United States; ^4^Department of Psychiatry, University of California, San Diego, San Diego, CA, United States

**Keywords:** independent component analysis, image-derived phenotype, machine learning, rsfMRI, group-level analysis

## Introduction

Independent Component Analysis (ICA) is a widely used, unsupervised, exploratory machine learning method (Comon, 1994) and is often applied to resting-state fMRI (rsfMRI) data (Nickerson et al., 2017). Though its usefulness is apparent, the most common applications of ICA involve substantial subjectivity. For example, the spatial components extracted through individual or group-level ICA are never identical between studies and are often labeled by visual inspection and expert opinion. The goal of this study was to establish a spatial component approach based on well-documented atlases derived from large-scale investigations. These components can subsequently be used in place of components tailored to fit each individual study's dataset.

The utility of ICA to extract meaningful functional connectivity patterns without the need for prior knowledge has been established by its application to large-scale studies like Human Connectome Project (HCP) and United Kingdom (UK) BioBank cohort (Miller et al., 2016; Smitha et al., 2017). Although ICA has been successfully applied to a wide range of applications in rsfMRI, there have long been some concerns about reproducibility and the subjectivity of ICA results (Friston, 1998). Also, ICA is a computationally-demanding approach, which may provide a barrier to researchers with limited resources or exceptionally large datasets. We present an approach that has the potential to substantially decrease both the subjectivity of ICA and its computational burden.

## The Critique on ICA

Performing ICA-based rsfMRI studies involves: data preprocessing and clean-up (sometimes through subject-level ICA or SICA), group-level ICA (GICA) on the entire dataset (usually with temporal concatenation), separating *signal* from *noise* independent components (ICs), network labeling, and time-series and spatial map extraction based on selected ICs for all subjects (Nickerson et al., 2017). Because ICA is a time-consuming and computationally resource-demanding procedure, a significant reduction in runtime may be worthwhile, especially in large-scale studies. Runtime issues aside, our main objective for improving this analysis pipeline focuses on producing objective, reproducible science. Furthermore, a main concern of the ICA pipeline lies in network labeling, where ICs representing potential resting-state networks (RSNs) of interest must be inspected by contextually-experienced brain anatomist(s) to be safeguarded against any misidentification. This limitation may challenge reproducibility of the results since this process could be quite arbitrary (Storti et al., 2013; Salimi-Khorshidi et al., 2014; Pruim et al., 2015). Also, running GICA on different datasets will not yield exactly the same components, and will output results that may not be closely matched with the results from other analyses (e.g., a single network may be split into 2 or 3 components, depending on the idiosyncrasies of the datasets used) (Wang and Li, 2015).

## Current Remedies

One approach to this problem is to utilize machine learning and deep learning solutions to compare the GICA results with established reference RSNs (Kozák et al., 2017; Zhao et al., 2018). Although this classification method categorizes the ICs objectively based on the provided template, replicability, and stability of this approach have yet to be benchmarked by large-scale studies and relies on large amounts of training data. Deep learning approaches involve additional concerns such as slow convergence and over-fitting, especially in MRI modalities (Srivastava et al., 2017).

Another method is “semi-blind ICA”, which uses prior knowledge in the form of a “template” that is entered at the beginning of each run of GICA to guide and improve estimation of network-related components (Lin et al., 2010). This method also requires well-established knowledge on the expected activation patterns in fMRI data, especially in task-based fMRI studies.

The alternative approach presented here may allow ICA pipelines to be more stable, faster and reproducible, in terms of extracting time-series of network(s) of interest from the subjects' data in a shorter time and with less computational resources. This is the case especially in time-constrained, resource-limited studies where access to experts for interpreting the GICA results may be a challenge.

## A Solution

We propose to use the ICs resulting from prior studies, such as the UK Biobank and HCP, in an “atlas-like” manner. Because such ICs are already published (Miller et al., 2016), they could be well-studied and agreed upon by the experts across the field. Following agreement, it would be possible to use the ICs as a reference to extract the time-series of subjects in matched groups, similar to an atlas, and interpret the ICs from other studies more objectively through automated, semi-automated, or conventional manual approaches.

**Figure 1:**
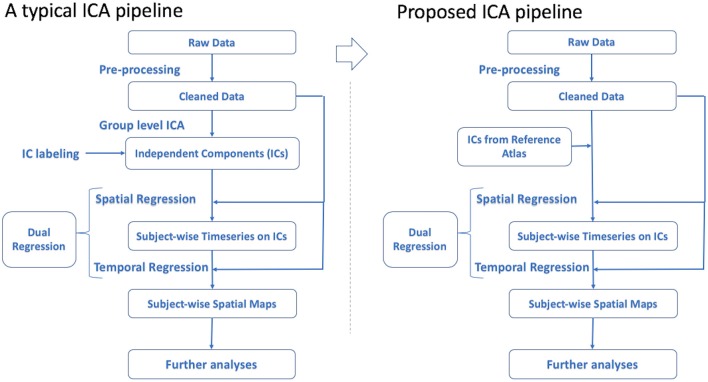
Conventional ICA pipeline vs. the proposed solution.

Results of GICA would have the potential be re-used in other studies (Bijsterbosch et al., 2017). The idea of using a reference in analyses is not novel. The use of references such as Montreal Neurological Institute (MNI) standard spaces or Harvard-Oxford cortical and subcortical structural atlases in preprocessing and analysis of imaging data is also based on the same concept of grouping data together to have a common frame of reference. This approach would be beneficial to ICA pipelines as well. This solution is depicted in Figure 1. In order to elaborate on this proposal, it is demonstrated by following the solution recommended by the widely-used FMRIB Software Library (FSL) package from Functional Magnetic Resonance Imaging Modeling (FMRIB) lab (Smith et al., 2004; Woolrich et al., 2009; Jenkinson et al., 2012).

After preprocessing the data, it is common to use cleaned data to perform GICA to detect ICs and then inspect the results to label and select RSNs (ICs) of interest. By means of dual regression, candidate ICs are mapped to each subject's functional data to extract subject-specific time-series and spatial maps of desired RSNs for use in subsequent analyses (Beckmann et al., 2009; Nickerson et al., 2017).

To alleviate the issues mentioned above, a more efficient approach would be to use previously labeled ICs from a large-scale study as an atlas of ICs to be applied in dual regression rather than performing GICA. This strategy reduces study-specific GICA to a regression method. As a candidate application for this improved approach, it would be possible to use the set of ICs from GICA obtained in one study to get the time-series of matched subjects in another demographically comparable study in a cross-site investigational manner. Also, investigating a subset group from the original dataset would be more efficient this way by eliminating the need to run GICA again on the subset. Although running GICA in this scenario may lead to a better model fit to the data this may not outweigh the benefit of having a more objective way of extracting the spatial maps and time-series. Also, using GICA can provide additional improvements of noise components, that would not be possible using a standard atlas. This may not be much of a concern, since sufficient preprocessing and noise removal can be performed at the individual subject level.

Atlas-based ICA would also benefit smaller-sized studies since the analysis pipeline would improve stability and allow results to be more readily comparable to other studies. In a conventional pipeline, the whole dataset must be preprocessed and ready before running GICA, therefore performing analysis on a subset before data collection is complete is not feasible and will not produce the same components as when the entire dataset is available. In addition, if a participant's data was excluded, re-running the GICA again would be necessary, instead of simply removing those records from the group analysis. The proposed approach would address these issues as well.

We tested our approach on an rsfMRI dataset from a recent within-subjects study (Le et al., 2018) with 20 individuals that completed three sessions of functional scans (60 scans in total). Following preprocessing we investigated the difference between the conventional ICA pipeline and our proposed pipeline and applied the GICA atlas from a subset of the Tulsa 1,000 study cohort (Victor et al., 2018) using a MacBook Pro machine with a 2.9 Ghz Intel Core i7 processor and 16 GB of memory. In the proposed pipeline, the time-series of the ICs for each subject (Stage 1 output from dual regression) were ready to use in 57 mins. Alternatively, performing GICA followed by dual regression in order to extract the time-series for each subject took 9 h and 33 min. The additional runtime required for running GICA is reasonable in this case, but would be of greater concern with a larger dataset. Additionally, GICA on the 60 scans produced a substantially different set of components, which makes it difficult or impossible to compute the same components as from the atlas.

Currently, there are published reference atlases on well-studied resting-state networks (Yeo et al., 2011). These atlases are useful for labeling the networks, yet they are binary network masks and lack components' voxel-wise weights, which are necessary for subject-wise time-course extraction. To the best of our knowledge, no atlas has been published with this information.

There are some limitations of this new proposed approach. Similar to other studies that use a common atlas, the subject population should be a reasonable representation of the subject group recruited in the reference studies. Since most large public datasets are from normal populations, one might question the practice of applying components derived from them to patient populations. This is a valid concern, which could be addressed by comparing GICA results from the target population with the standard atlas components. Similarly, there may be systematic differences introduced by machine types and scanning parameters, which warrants a thorough investigation. Regardless, it would be surprising if factors such as scanning parameters impacted large-scale functional organization of the brain. Also, across the field there is no standard procedure for preprocessing the data, so careful consideration must be taken when applying an IC atlas and preprocessing procedures must be compatible (Wetherill et al., 2018), as different preprocessing methods may result in a different set of outcomes. In addition, performing preprocessing on the data before comparing the results to IC atlas is necessary. For example, if an atlas uses 2 mm resolution maps, applying it to studies with different set of rules would require auxiliary processing steps.

To the best of our knowledge, there are several ICA results published and available in the literature (Beckmann et al., 2005; Smith et al., 2009; Laird et al., 2011) including from the UK Biobank cohort (Miller et al., 2016). Such references have the potential to be applied as “Atlases of ICs” on other studies, but only with extensive documentation. Publishing group-level ICA results and documenting them in an atlas-like manner would also allow researchers to keep their (testing) data separate from the training data used to build the models, which is another consideration that has garnered increased attention recently (Scheinost et al., 2019), along with the need for more open, externally validated science.

## Author Contributions

RK proposed the research idea and revised the manuscript. MM performed the research and authored the manuscript. HE developed the idea and revised the manuscript. TV provided the datasets and revised the manuscript. MP supervised the project and revised the manuscript and provided feedback on the research and the manuscript.

### Conflict of Interest

MP is an advisor to Spring Care, Inc., a behavioral health start up, he has received royalties for an article about methamphetamine in UpToDate. The remaining authors declare that the research was conducted in the absence of any commercial or financial relationships that could be construed as a potential conflict of interest.
